# Annotations of Lung Abnormalities in Shenzhen Chest X-ray Dataset for Computer-Aided Screening of Pulmonary Diseases

**DOI:** 10.3390/data7070095

**Published:** 2022-07-13

**Authors:** Feng Yang, Pu-Xuan Lu, Min Deng, Yì Xiáng J. Wáng, Sivaramakrishnan Rajaraman, Zhiyun Xue, Les R. Folio, Sameer K. Antani, Stefan Jaeger

**Affiliations:** 1 National Library of Medicine, National Institutes of Health, Bethesda, Maryland, USA; 2 Department of Radiology, Shenzhen Center for Chronic Disease Control, Shenzhen, China; 3 Department of Imaging and Interventional Radiology, Faculty of Medicine, The Chinese University of Hong Kong, Prince of Wales Hospital, N.T., Hong Kong; 4 Diagnostic Imaging & Interventional Radiology, Moffitt Cancer Center, Tampa, Florida, United States

**Keywords:** Tuberculosis (TB), annotations, abnormalities, computer-aided diagnosis, chest X-ray (CXR) images

## Abstract

Developments in deep learning techniques have led to significant advances in automated abnormality detection in radiological images and paved the way for their potential use in computer-aided diagnosis (CAD) systems. However, the development of CAD systems for pulmonary tuberculosis (TB) diagnosis is hampered by the lack of training data that is of good visual and diagnostic quality, of sufficient size, variety, and, where relevant, containing fine region annotations. This study presents a collection of annotations/segmentations of pulmonary radiological manifestations that are consistent with TB in the publicly available and widely used Shenzhen chest X-ray (CXR) dataset made available by the U.S. National Library of Medicine and obtained via a research collaboration with No. 3. People’s Hospital Shenzhen, China. The goal of releasing these annotations is to advance the state-of-the-art for image segmentation methods toward improving the performance of fine-grained segmentation of TB-consistent findings in digital Chest X-ray images. The annotation collection comprises the following: 1) annotation files in JSON (JavaScript Object Notation) format that indicate locations and shapes of 19 lung pattern abnormalities for 336 TB patients; 2) mask files saved in PNG format for each abnormality per TB patient; 3) a CSV (comma-separated values) file that summarizes lung abnormality types and numbers per TB patient. To the best of our knowledge, this is the first collection of pixel-level annotations of TB-consistent findings in CXRs.

**Dataset:** https://data.lhncbc.nlm.nih.gov/public/Tuberculosis-Chest-X-ray-Datasets/Shenzhen-Hospital-CXR-Set/Annotations/index.html.

## 1. Introduction

Tuberculosis (TB) is the second leading mortality-causing infectious disease after COVID-19 [1]. There is a large, persistent gap in global TB case detection which has been exacerbated due to reduced access to screening, diagnostic and treatment caused by the COVID-19 pandemic. In 2020, an estimated 10 million people fell ill with TB globally, but only 5.8 million of these people were diagnosed and reported [1]. Chest X-ray (CXR) is a recommended and widely-used tool for TB screening [2], however, its effectiveness in resource-constrained settings is restricted by limited specificity and lack of access to sufficiently trained radiologists [3]. The development of new hardware (such as GPUs) and software techniques present an opportunity to improve computer-aided diagnostic systems for TB identification and lung abnormality detection. However, progress in the field has been hampered by the lack of publicly available radiographs, especially fine-grained abnormality annotations, which are important for training and evaluating of machine learning algorithms used in computer-aided diagnostic systems [4]. The U.S. National Library of Medicine (NLM) has made the Shenzhen and Montgomery County CXR datasets publicly available^1^ [5], which in addition to a subject’s TB status (i.e., positive or negative/normal) also includes metadata for age and gender. The TB cases have been either confirmed microbiologically, or when this was not possible, confirmed by clinical symptoms and imaging appearances consistent with TB, including a positive response to anti-TB medication, and excluding other causes.

We further this effort by collecting and annotating lung abnormalities for TB patients on pixel level (fine-grained) for the Shenzhen CXR dataset and making the annotations available to the public to help advance research in fine-grained segmentation of TB-consistent findings as well as reduction of false positives and false negatives from deep learning models. To the best of our knowledge, unlike other collections that provide coarse bounding-box annotations [6], this is the only collection of pixel-level annotations of TB-consistent findings in CXRs. As mentioned in [5], the dataset was exempted from IRB review at the collecting institution. At NIH, the dataset use and public release were exempted from IRB review by the NIH Office of Human Research Projections Programs (OHRP # 5357). In the following section, we will describe in detail the annotations of lung abnormalities for TB patients, which consist of three main parts: 1) annotation files in JSON (JavaScript Object Notation) format that indicate the type, location, and shape of 19 abnormalities for TB patients; 2) binary mask image files saved in PNG format for each lung abnormality per patient; 3) a CSV (comma-separated values) file that summarizes abnormality types and numbers for each TB CXR image.

## 2. Annotations of lung abnormalities for TB patients in Shenzhen CXR dataset

The annotations of lung abnormalities for TB patients in the Shenzhen dataset were collected in collaboration with radiologists at the Chinese University of Hong Kong, China. The Shenzhen CXR dataset includes 662 CXRs, of which 326 are normal cases and 336 are cases with manifestations of TB [5]. The abnormality annotations were performed on the 336 TB CXRs by two radiologists from the Chinese University of Hong Kong. The labeling was initially conducted by a junior radiologist (M.D.), then labels were all checked by a senior radiologist (Y.X.J.W.), with consensus reached for all cases. The abnormalities are initially annotated using the Firefly labeling tool [7] with polygon points and saved in TXT format for 19 abnormal categories including: pleural effusion, apical thickening, single nodule (non-calcified), pleural thickening (non-apical), calcified nodule, small infiltrate (non-linear), cavity, linear density, severe infiltrate (consolidation), thickening of the interlobar fissure, clustered nodule (2mm-5mm apart), moderate infiltrate (non-linear), adenopathy, calcification (other than nodule and lymph node), calcified lymph node, miliary TB, retraction, other, and unknown. For better visualization and easier data interchange, we convert the annotations from TXT format to JSON format. Binary masks for abnormal areas are also generated for each TB CXR image.

Of note, since the JSON files will be publicly available, they could be used as ground truth or comparison in future studies and hackathons as was done recently with a similar set [8].

### 2.1. Annotations in JSON format and visualization

**Figure 1.**
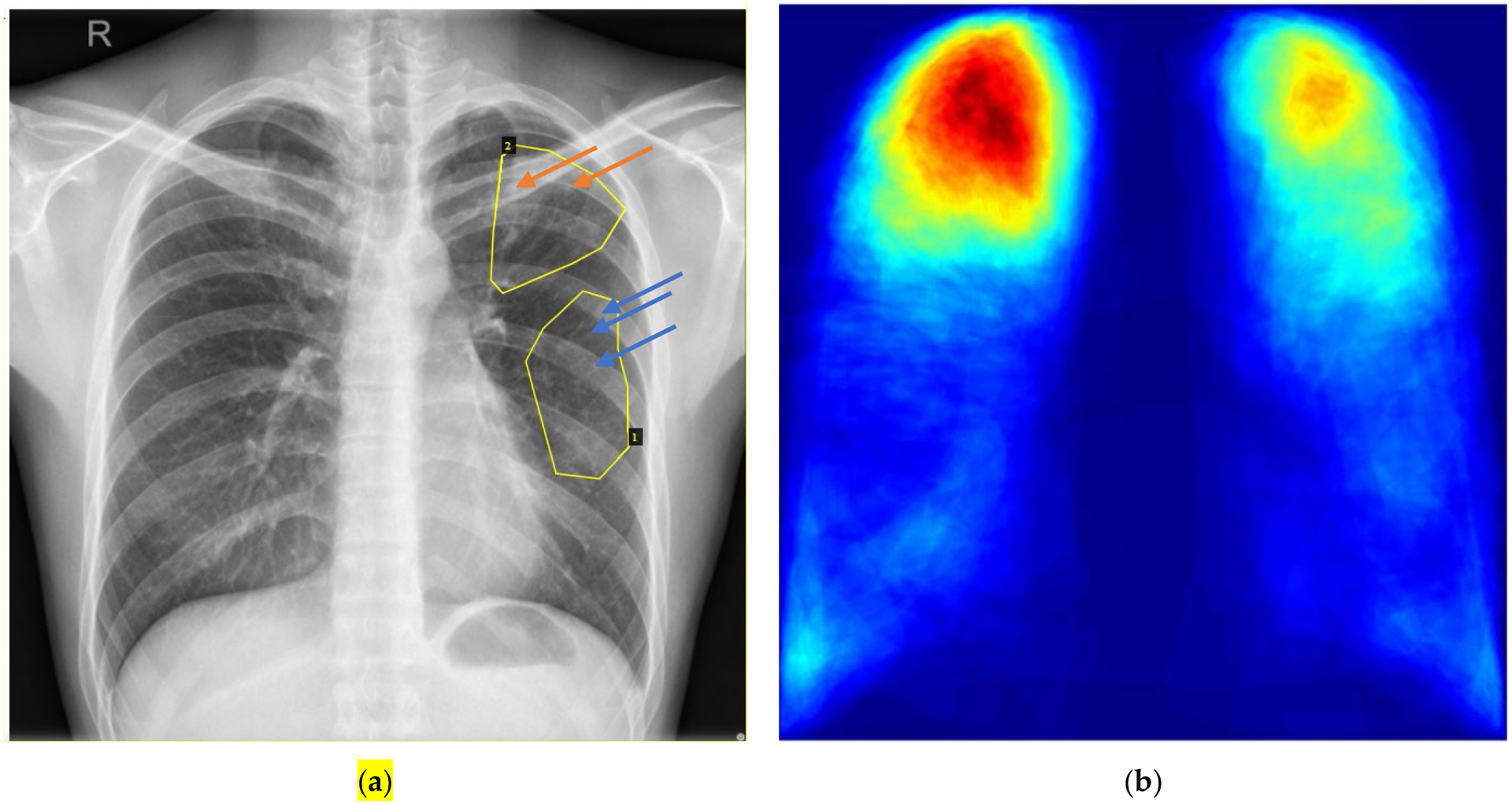
Annotation visualization and a representative heatmap generated from all annotations. (a) Visualization of annotations for CHNCXR_0327_1.png by VIA^2^. Two steps are needed to visualize annotations in JSON format: 1) load a CXR image via “Add Files” in the left column of the webpage; 2) load the corresponding annotation with “Annotation->Import Annotations (from json)” from the top bar. There are two abnormal areas in this CXR image. Their types are shown when clicking with the mouse on their corresponding areas. Area 1 includes clustered nodules (2mm-5mm apart) (indicated by blue arrows) and Area 2 is with calcified nodules (indicated by orange arrows). (b) Representative heatmap of all 19 finding categories compatible with TB. It is of no surprise to clinicians that the right upper lobe is most involved (due to the more vertical nature of the bronchus intermedius on the right relative to the left mainstem bronchus), followed by left upper lobe. Similar right sided predominance is supported by increased involvement of right costophrenic angle and supportive findings expected with TB distribution.

An annotation file for a given image has the same name as the CXR image, except that the extension of “png” is replaced with “json”. It includes the following information: filename, image size, abnormality shape (polygon), *x* coordinates for all points, *y* coordinates for all points, and abnormality type. An annotation file in JSON format can be directly visualized by VGG Image Annotator (VIA) [9], a web-browser-based annotation tool, by loading both a CXR image and a corresponding annotation file. Figure 1a shows an example of visualizing annotations for a given image with VIA. An all-in-one annotation file for 336 CXR images, named Annotations_AllinOne_json.json, is also generated to avoid loading annotation files one-by-one into VIA. See representative distribution of annotated TB findings in Figure 1b.

### 2.2. Binary abnormality masks

All mask file names follow the same template: CHNCXR_####_1_****_X.png, where CHNCXR_####_1 is the name of an original CXR PNG image with #### representing a 4-digit numerical identifier and 1 indicating an abnormal CXR image; **** is the type of abnormality, and X ranges from 1 to 19, indicating the mask ID. For a given CXR image CHNCXR_####_1.png including M abnormalities, there will be a total of M masks generated and saved separately in PNG format. Taking the CXR image CHNCXR_0329_1.png as an example, two abnormalities are found: clustered nodule (2mm-5mm apart) and calcified nodule; therefore, two masks are generated with the following names:

CHNCXR_0329_1_Clustered_Nodule_(2mm-5mm_apart)_1.png
CHNCXR_0329_1_Calcified_Nodule_2.png.

Within the 336 abnormal CXRs, radiological signs of TB are observed only in 330 CXRs. The six CXRs with no radiological signs are CHNCXR_0467_1.png, CHNCXR_0484_1.png, CHNCXR_0606_1.png, CHNCXR_0609_1.png, CHNCXR_0612_1.png, and CHNCXR_0624_1.png. No marks or annotations are generated for these six CXR images.

### 2.3. CSV file

The CSV file named “Statistics_ShenzhenDataset.csv” provides information on abnormality type and number of occurrences for each TB CXR image. It includes 20 columns, where the first column is the CXR image name, and columns 2 to 20 correspond to the 19 abnormalities. Taking the CXR image CHNCXR_0329_1.png as an example again, both columns “Calcified_Nodule” and “Clustered_Nodule_(2mm-5mm_apart)” are assigned 1s, indicating that one calcified nodule and one clustered nodule (2mm-5mm apart) are found in this CXR image. Table 1 shows the total number of annotations per category for 336 TB CXRs in the Shenzhen dataset.

## 3. Summary

In this paper, we establish a collection of annotations/segmentations for lung abnormalities in the publicly available Shenzhen chest X-ray (CXR) dataset [1], which enables training of deep learning models for TB diagnosis and is expected to improve fine-grained segmentation of TB-consistent findings and reduce false positives and false negatives for deep learning models. This is the first collection of pixel-level annotations of TB-consistent findings in CXRs.

## Figures and Tables

**Table 1.** Summary of the total number of annotations per abnormality category for 336 TB CXRs in the Shenzhen dataset.

Abnormality type	Total number	Abnormality type	Total number
Pleural effusion	59	Clustered nodule (2mm-5mm apart)	146
Apical thickening	57	Linear density	138
Single nodule (non-calcified)	130	Adenopathy	21
Pleural thickening (non-apical)	49	Calcification (other than nodule and lymph node)	19
Calcified nodule	79	Calcified lymph node	2
Small infiltrate (non-linear)	163	Miliary TB	6
Moderate infiltrate (non-linear)	147	Retraction	10
Severe infiltrate (consolidation)	35	Other	18
Cavity	45	Unknown	14
Thickening of the interlobar fissure	15		

## Data Availability

The annotation data presented in this study is now openly available on: https://data.lhncbc.nlm.nih.gov/public/Tuberculosis-Chest-X-ray-Datasets/Shenzhen-Hospital-CXR-Set/Annotations/index.html.

## Footnotes

1
https://data.lhncbc.nlm.nih.gov/public/Tuberculosis-Chest-X-ray-Datasets/Shenzhen-Hospital-CXR-Set/index.html

2
https://www.robots.ox.ac.uk/~vgg/software/via/via.html
